# Palliative Non-Operative Management in Geriatric Hip Fracture Patients: When Would Surgeons Abstain from Surgery?

**DOI:** 10.3390/jcm13061594

**Published:** 2024-03-11

**Authors:** Michael Bui, Catharina G. M. Groothuis-Oudshoorn, Annemieke Witteveen, Johannes H. Hegeman

**Affiliations:** 1 Department of Health Technology and Services Research, Technical Medical Centre, University of Twente, 7500 AE Enschede, The Netherlands; c.g.m.oudshoorn@utwente.nl; 2 Department of Biomedical Signals and Systems, Technical Medical Centre, University of Twente, 7500 AE Enschede, The Netherlands; a.witteveen@utwente.nl; 3 Department of Surgery, Ziekenhuisgroep Twente, 7609 PP Almelo, The Netherlands

**Keywords:** hip fractures, geriatrics, frailty, palliative non-operative management, decision-making, conjoint analysis, structured expert judgement

## Abstract

**Background:** For hip fracture patients with a limited life expectancy, operative and palliative non-operative management (P-NOM) can yield similar quality of life outcomes. However, evidence on when to abstain from surgery is lacking. The aim of this study was to quantify the influence of patient characteristics on surgeons’ decisions to recommend P-NOM. **Methods:** Dutch surgical residents and orthopaedic trauma surgeons were enrolled in a conjoint analysis and structured expert judgement (SEJ). The participants assessed 16 patient cases comprising 10 clinically relevant characteristics. For each case, they recommended either surgery or P-NOM and estimated the 30-day postoperative mortality risk. Treatment recommendations were analysed using Bayesian logistic regression, and perceived risks were pooled with equal and performance-based weights using Cooke’s Classical Model. **Results:** The conjoint analysis and SEJ were completed by 14 and 9 participants, respectively. Participants were more likely to recommend P-NOM to patients with metastatic carcinomas (OR: 4.42, CrI: 2.14–8.95), severe heart failure (OR: 4.05, CrI: 1.89–8.29), end-stage renal failure (OR: 3.54, CrI: 1.76–7.35) and dementia (OR: 3.35, CrI: 1.70–7.06). The patient receiving the most P-NOM recommendations (12/14) had a pooled perceived risk of 30-day mortality between 50.8 and 62.7%. **Conclusions:** Overall, comorbidities had the strongest influence on participants’ decisions to recommend P-NOM. Nevertheless, practice variation and heterogeneity in risk perceptions were substantial. Hence, more decision support for considering P-NOM is needed.

## 1. Introduction

In worldwide practice, operative treatment is considered to be superior over non-operative management in terms of clinical outcomes for the majority of hip fracture patients [1,2]. It is well established that the mortality rate is significantly higher in non-operatively treated patients than in operatively treated patients [3,4,5]. However, in the case of frail older adults with a limited life expectancy, surgeons have started to question the superiority of surgery [6,7]. Clinical guidelines often focus on functional recovery to pre-fracture levels [8], while patients with a limited life expectancy might prioritise their quality of life (QoL) instead [9]. In these cases, surgical overtreatment should be avoided due to its negative repercussions on patients and families, which include iatrogenesis and anxiety [10,11]. Hence, there is increasing awareness that palliative non-operative management (P-NOM) should be considered as a valid care option amongst frail older adults [7,9,12,13,14,15].

Particularly amongst patients of advanced age with multiple physical and cognitive comorbidities, there is a pressing need for counselling regarding survival prognoses and advance care planning [16]. By properly informing frail patients on the available treatment options and examining how these align with their goals of care through shared decision-making (SDM) [17], patients and clinicians might come to the conclusion that P-NOM is preferred. Affirmatively, a single-centre retrospective cohort study found that the percentage of patients electing P-NOM increased significantly over the years (2.7% vs. 9.1%) after implementing comprehensive geriatric assessments with SDM [18]. Still, uncertainties regarding the optimal treatment choice might persist during SDM in complex patient cases [7]. A paucity of decision support for P-NOM in current clinical guidelines poses challenges for the preoperative decision-making process. Therefore, more evidence regarding the choice between surgery and P-NOM is required to optimise treatment plans for frail older adults.

Only a few studies have thus far investigated the motives behind electing P-NOM. In most cases, P-NOM was preferred when early mortality or other poor prognoses were anticipated for operative treatment due to frailty, for example, caused by comorbidities, poor functional status and declining cognitive functioning [18,19]. While these attributes could be used to identify patients who would not benefit from operative treatment, it remains a challenging task. Various prediction models for 30-day mortality following hip fracture surgery have been developed to identify patients who are unfit for operative treatment [20,21,22,23,24]. However, these models showcased moderate discriminative ability, making them premature for clinical practice. When data-driven approaches are not sufficiently reliable, domain experts should be consulted [25,26]. The synthesis of clinicians’ treatment preferences for various patient cases aids in understanding which specific patients would benefit from which treatments [27].

The current study proposes a clinical vignette methodology to elicit and analyse surgical residents’ and orthopaedic trauma surgeons’ treatment preferences for frail older hip fracture patients with limited life expectancy. This is a type of conjoint analysis (CA) [28,29], in which the decision-making behaviours of medical experts are studied in various scenarios known as vignettes [30]. A vignette is defined as “a short, carefully constructed description of a person, object, or situation, representing a systematic combination of characteristics” [31] (p. 128). Given that clinicians’ judgements of vignettes and their responses to real-life cases are sufficiently congruent [32], clinical vignette studies provide a means to reliably simulate and analyse complex decision-making processes in healthcare. The insights gained facilitate the understanding of which factors are influential in decision-making for surgeons and help inform clinical practices and policy development to support decision-making [33].

While individual patient attributes may influence physicians’ treatment preferences, they may also shape their overall perception of patients’ early mortality risks. Capturing early mortality risk assessments is pertinent since they could influence the likelihood of considering P-NOM [6,7,8]. Therefore, the current study proposes to additionally elicit the perceived risks of 30-day postoperative death for each vignette through a structured expert judgement (SEJ) [34].

To support preoperative decision-making for frail hip fracture patients with a limited life expectancy, it is imperative to understand how patient characteristics and mortality risk perceptions affect treatment decisions. Hence, the aim of this exploratory study is to conduct a clinical vignette study and SEJ to systematically capture the expertise of surgical residents and orthopaedic trauma surgeons to synthesise recommendations for clinical guidelines. To the best of our knowledge, we are the first to conduct a clinical vignette study and SEJ to examine preoperative decision-making for frail hip fracture patients.

## 2. Materials and Methods

### 2.1. Data Collection

The clinical vignette study and SEJ were distributed to surgical residents and orthopaedic trauma surgeons from three Dutch hospitals through an online survey between June and August 2022. Participants were approached through an e-mail explaining the purpose of the study along with a link to the survey.

### 2.2. Selection of Patient Attributes and Levels for the Vignettes

Predictors for early mortality were chosen as primary attributes for the design of the vignettes, since P-NOM was mostly reserved for patients with a limited life expectancy [8,9]. In our previous work [35], we conducted a systematic review and meta-analysis to identify these predictors. To analyse participants’ decision-making behaviours as comprehensively as possible, the vignettes were designed using the maximum number of attributes recommended in practice, that is, 10 attributes [33].

All high-quality evidence predictors for 30-day mortality identified in our meta-analysis were selected as attributes for the vignettes (age, gender, ASA classification [36], institutional residence and metastatic cancer). Amongst the moderate-quality evidence predictors, only those for which confidence in the existence of a true significant association with mortality was expressed were selected (dementia, renal failure and heart failure). To increase ecological validity, functional status was included, as guidelines for preoperative decision-making are centred around functional recovery [9]. Finally, to enforce applicability to the study population of interest, fracture type was selected as an attribute.

When constructing vignettes, implausible combinations of attribute levels should be avoided. Amongst the chosen attributes, implausibility concerns were raised for the ASA score. Since ASA scores increase with the severity of diseases, not all pairs of comorbidities and ASA scores would be logical to present simultaneously in the vignettes. Hence, attribute levels of comorbidities were defined such that they were maximally compatible with all ASA attribute levels chosen in this study. To keep the total number of vignettes low, the number of attribute levels was mostly restricted to two. Since a dichotomy of health conditions and functional statuses could potentially be too coarse to inform decision-making, the vignettes were pilot tested with a surgical resident and orthopaedic trauma surgeon. Both physicians agreed that it was not necessary to introduce additional attribute levels. An overview of the attribute levels along with the rationale behind the chosen definitions is depicted in Table 1.

### 2.3. Experimental Design of Patient Vignettes

The 10 attributes yielded a full factorial design comprising 2^9^ × 3 = 1536 vignettes. However, one attribute level combination was deemed implausible: ASA III paired with metastatic cancer [45]. Hence, all vignettes containing this combination were removed from the full factorial design to reduce measurement errors [46], leaving a total of 1152 vignettes. As it was not feasible to present all 1152 vignettes to each participant, a D-optimal main effects design [47] was generated from this subset with R version 4.0.2 using the skpr package [48]. Through experimental designs, smaller subsets of vignettes can be selected while safeguarding the precision and unbiasedness of the statistical analysis [49]. The number of vignettes was minimised by inspecting the relative gain in D-efficiency upon increasing the number of vignettes over a range of 12 to 24. Based on these trials, a design comprising 16 vignettes with a D-efficiency of 94.4% was chosen. The full experimental design can be found in Table A1 of Appendix A.

### 2.4. Survey Design

The survey consisted of four sections. The first section covered background questions about participants’ medical professional status and years of working experience. In the second section, they were presented with the vignettes. For each vignette, they were asked to (1) recommend either surgery or P-NOM, (2) rate how certain they were about the optimality of their recommendation on a 5-point Likert scale and (3) estimate the probability of 30-day postoperative mortality. Whenever they elected operative treatment, they were asked whether the treatment intentions were palliative or curative. In the third section, participants answered 14 SEJ questions to assess their expertise in mortality prediction. Finally, participants gave feedback on information they missed in the vignettes.

### 2.5. Elicitation and Analysis of Treatment Preferences

The aim of the vignette study was to quantify the average impact of patient attributes on participants’ treatment preferences in terms of odds ratios (ORs). ORs were estimated using a hierarchical Bayesian logit with random intercepts, in which treatment choices were regressed against the attributes in the vignettes. To examine the degree to which treatment recommendations could be explained by participants’ personal preferences rather than changes in attribute levels, the intraclass correlation coefficient (ICC) was computed.

A Bayesian estimation framework was chosen for the main analysis since a low response rate to the survey was anticipated. When prior knowledge about the effect sizes of the individual attributes is available, Bayesian models can still provide valid regression outcomes despite small sample sizes [50,51,52,53,54]. Since early mortality risk is the primary reason for electing P-NOM [8], we assumed that the attributes’ prognostic values for death could be seen as proxies for participants’ inclinations to choose P-NOM. Therefore, we primarily used our systematic review and meta-analysis for predictors of early mortality to derive prior knowledge on the effect sizes and uncertainties around the beta coefficients of the Bayesian model (see Table 2). We followed the When-to-Worry-and-How-to-Avoid-the-Misuse-of-Bayesian-Statistics checklist to ensure methodological rigour [55].

The posterior distributions for each regression coefficient were estimated via Markov Chain Monte Carlo (MCMC) sampling [60]. For this, 15,000 posterior samples were drawn after a burn-in phase of 1000 samples. Point estimates were obtained by computing the posterior means. The model was implemented in R version 4.0.2, using the MCMCpack package [61].

### 2.6. Bayesian Convergence Diagnostics and Sensitivity Analysis

To ensure that the regression coefficients had converged to stable estimates, several diagnostic tests were conducted. First, trace and autocorrelation plots were inspected for MCMC convergence. The stationarity of the Markov chains was assessed with Geweke’s convergence test [62]. Second, to determine whether the resulting posteriors were sufficiently smooth, histograms of the posterior draws were inspected.

Finally, to assess the extent to which our prior beliefs affected the ORs, the regression analysis was re-evaluated with noninformative priors, that is, N(0, 2), which neither favoured P-NOM nor surgery. The influence of priors was considered (1) small if the relative deviation (RD) was at most 10% and the substantive results remained the same, (2) moderate if 10% < RD ≤ 20% and the substantive results remained the same and (3) large otherwise.

RD = 100% × |OR_informative_ − OR_uninformative_|/OR_informative_ (1)

### 2.7. A Priori Power Analysis and Sample Size Calculations

**Figure 1:**
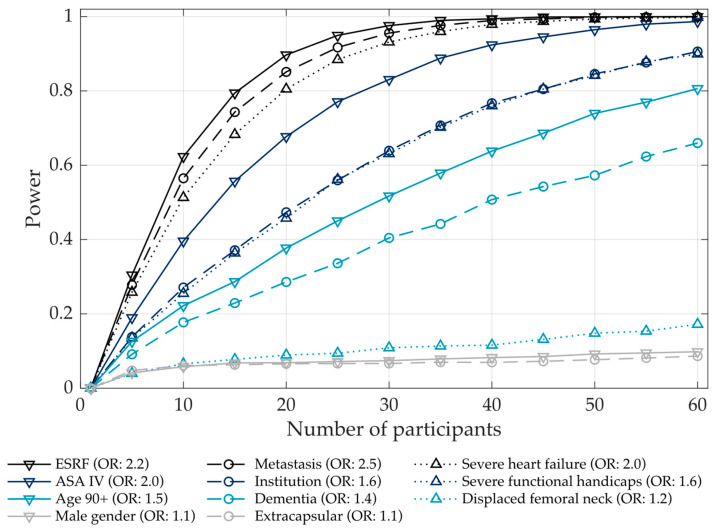
Power curves for the attribute levels used in the vignettes. The respective odds ratios (ORs), which were assumed during the power calculations, are listed behind each attribute level.

Health policy recommendations based on non-significant outcomes should not be made without considering whether the study had sufficient power to detect small yet meaningful effects [63]. Therefore, an a priori power analysis was conducted using 10,000 Monte Carlo simulations [64]. For simplicity, a logit model was used as an analytical outcome model in the simulations to obtain a rough estimate for the required sample size. The resulting power curves shown in Figure 1 indicated that approximately 55 participants were required to attain a power above 60% for 8/11 attribute levels.

### 2.8. Elicitation and Analysis of Risk Perceptions

The goal of the SEJ was to elicit and aggregate participants’ 30-day mortality risk perceptions of frail geriatric patients undergoing hip fracture surgery. Expert elicitation was performed using Cooke’s Classical Model for SEJs [34]. The Classical Model enforces empirical control by first scoring how statistically accurate and informative participants are in the estimation of verifiable variables, prior to aggregating their judgements on unknown variables. Participants with higher scores are assigned higher performance-based weights in the aggregation to obtain the best estimate of the unknown target variable.

Calibration questions were used to measure participants’ performances. In this case, calibration questions referred to verifiable questions about 30-day mortality prevalence percentages amongst subpopulations of hip fracture patients. Participants are not expected to know the exact percentages but should be able to capture them reliably based on their expertise by defining adequate credible intervals (CrIs). The 5th, 50th and 95th percentiles (q_5_, q_50_ and q_95_) were chosen for CrI elicitation, as this is the most common practice in SEJs [56,65]. Through such 90% CrIs, participants express their beliefs that there is a 90% chance that the true mortality rate falls between q_5_ and q_95_.

### 2.9. Structured Expert Judgement Instruments

For each vignette, the following target question was posed: “According to you, what is the probability that a patient with these characteristics would die within 30 days after hip fracture surgery?” Participants were asked to choose a probability bin from the set (0–0.1), (0.1–0.2), …, (0.9–1.0), which reflected their beliefs best. The middle value of each bin functioned as a point estimate for pooling later in the analysis.

Calibration questions were based on 30-day mortality data from the Dutch Hip Fracture Audit Taskforce Indicators (DHFA-TFI) group [57], which described a total of 7506 patients. To ensure similarity with the target questions, calibration questions were based on patient subgroups, which resembled the vignettes. Similarity was ensured through age matching (≥80 years) and choosing overlapping attributes: gender, fracture type, dementia, functional status in ADL, ASA scores and institutional residence. Since these characteristics were insufficient to construct ample diverse calibration questions, mobility, malnutrition and anaemia were included as additional attributes. An example of a calibration question is: “How many percent of the hip fracture patients aged **90 years or older**, who were **mobile without walking aids** and **did not have dementia**, died within 30 days following hip fracture surgery between 2017 and 2019, according to the DHFA-TFI group?” All 14 calibration questions can be found in Table A2 of Appendix A.

The ground truth of the calibration questions could not be obtained directly from the DHFA-TFI cohort since there were missing data. Information on 30-day mortality was missing for 19.5% of the 7506 patients. Missing entries were imputed with Multiple Imputation by Chained Equations (MICE) [58]. MICE was used to create 20 imputed data sets [58], from which the 30-day mortality percentages were extracted and pooled. With the true mortality rates available, participants’ performances could be measured using two scoring metrics: the calibration score and the information score.

### 2.10. Expert Scoring and Performance Weighting

The calibration score evaluated the statistical accuracy of participants’ CrIs. Calibration was measured by examining how well they captured the true 30-day mortality rates across the four inter-percentile ranges: <q_5_, (q_5_–q_50_), (q_50_–q_95_) and >q_95_. Participants were said to be well-calibrated if their 90% CrIs captured the true 30-day mortality rates across 90% of the calibration questions, such that the true values fell below q_5_ in 5% of the cases, between q_5_ and q_50_ in 45% of the cases, between q_50_ and q_95_ in 45% of the cases, and above q_95_ in 5% of the cases. The calibration score was defined as the p-value of a Chi-squared test examining whether the CrIs indeed captured the true mortality rates according to this theoretical distribution. A calibration score of 1 indicated the highest level of statistical accuracy.

The information score indicated the degree to which participants deemed some values more likely to be true than others. As they could in theory achieve perfect statistical accuracy by specifying overly wide CrIs, the information score was introduced to compensate for this. Participants received higher information scores if they specified more concentrated CrIs. The computational steps are described in more detail elsewhere [59].

Finally, the calibration scores and information scores were multiplied for each participant to obtain an overall performance weight. The weights were then normalised, such that they summed to 1 across all participants. For each vignette, the estimated probabilities of 30-day mortality were then combined into a performance-weighted average. Pooling with equal weights was performed as a sensitivity analysis.

## 3. Results

### 3.1. Respondents

In total, 21 survey responses were collected. These included 14 complete responses for the clinical vignette study (6 orthopaedic trauma surgeons and 8 surgical residents), of which 9 were also complete for the SEJ (4 orthopaedic trauma surgeons and 5 surgical residents). The medians and interquartile ranges of years of experience for orthopaedic trauma surgeons and surgical residents were 11.3 (8.5–18.1) and 4.0 (2.8–5.0), respectively.

### 3.2. Results of the Vignette Study

Table 3 depicts the outcomes of the vignette study. Amongst the inspected patient attributes, only four showcased 95% CrIs, which did not overlap with the null effect. In descending order of effect size, these were metastatic carcinoma (OR: 4.42, 95% CrI: 2.14–8.95), severe heart failure (OR: 4.05, 95% CrI: 1.89–8.29), end-stage renal failure (OR: 3.54, 95% CrI: 1.76–7.35) and dementia (OR: 3.35, 95% CrI: 1.70–7.06). From the estimated ORs, comorbid conditions appeared to affect the likelihood of recommending P-NOM the most. For instance, the odds that patients with metastatic carcinomas received a P-NOM recommendation were 4.42 times higher than for patients without metastatic carcinomas.

For all regression coefficients, the diagnostic tests indicated that the estimates were stable (see Appendix B). Furthermore, all substantive conclusions, that is, whether the CrIs were non-overlapping with the null effect, were robust with respect to the decreased informativeness of priors. The sensitivity analysis showed that the informative priors had little influence on the ORs of end-stage renal failure, preoperative residence, functional status, gender and age. The prior influence was moderate for severe heart failure, dementia and fracture type. Finally, priors were highly influential for the effect estimates of metastatic carcinoma and physical status.

### 3.3. Results of the Structured Expert Judgement

**Figure 2:**
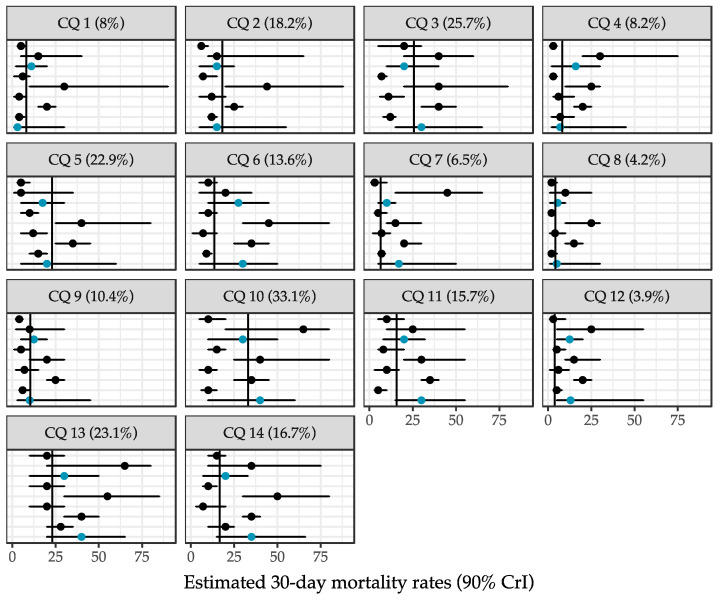
Overview of participants’ responses to the 14 calibration questions. The dots represent participants’ best estimates of the 30-day mortality rates and the horizontal bars represent their 90% credible intervals. The true 30-day mortality rate is reported in parentheses and depicted by the vertical line. Participants with calibration scores above 0.05 are highlighted in blue.

Figure 2 depicts the responses to the 14 calibration questions of nine participants who completed the SEJ. Only two participants (surgical residents) managed to achieve calibration scores above 0.05, indicating that they were well-calibrated. With calibration scores of 0.53 and 0.32, their judgements accounted for a cumulative normalised weight of 93% in the performance-weighted pooled estimates.

**Figure 3:**
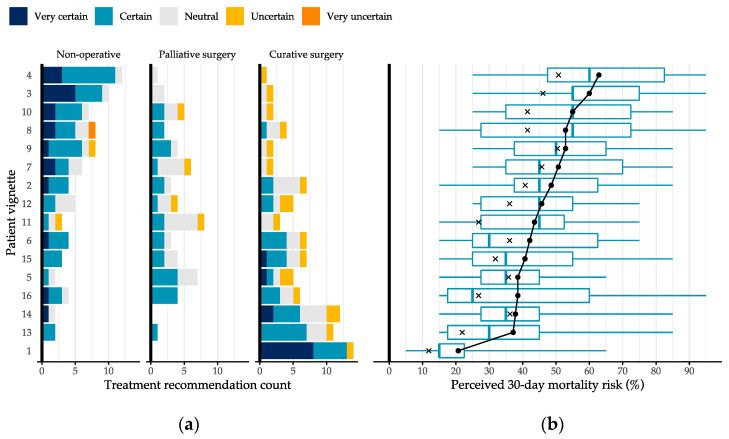
Distribution of participants’ responses to the 16 vignettes. Vignettes were sorted in descending order of mean 30-day mortality risk. (**a**) Overview of recommended treatments per vignette, subcategorised by participants’ confidence in the optimality of the elected treatment. (**b**) Boxplots of the estimated 30-day mortality risks per vignette. Circles denote the mean probabilities (equal weights) and crosses denote the performance-weighted pooled estimates.

An overview of the 30-day mortality probability estimates obtained through linear opinion pooling with equal weights and performance-based weights is shown in Figure 3b. For each vignette, the performance-based weights estimates were consistently lower than the equal weights estimates. This entailed that those who had high calibration scores, estimated lower mortality risks than the average participants in the study sample. The pooled probabilities across all vignettes ranged between 20.7–62.7% and 11.9–50.8% for equal and performance-based weights, respectively.

### 3.4. Heterogeneity in Treatment Preferences and Risk Perceptions

The trends shown in Figure 3 indicated that P-NOM was more frequently recommended to patients for whom a higher 30-day mortality risk was prognosticated on average. However, preferences for P-NOM differed considerably from participant to participant. The ICC was estimated at 0.299, which entailed that personal differences between participants explained 29.9% of the treatment recommendations. The apparent preference heterogeneity can be further exemplified by examining the P-NOM choice proportions across individual participants (see Table 4). On the highest extreme, four orthopaedic trauma surgeons each recommended P-NOM 10–12 times. On the lowest extreme, four surgical residents each recommended P-NOM 0–3 times. Hence, although participants expressed low degrees of uncertainty in the optimality of their elected treatments (see Figure 3a), their recommendations were divided.

Furthermore, as shown in Figure 3b, participants’ perceptions of 30-day mortality risks were also highly dispersed. For vignettes 8 and 16, for instance, the estimated prognoses varied between 15 and 95%. On the individual participant level, a difference in how sensitive their risk perceptions were to changes in patient attributes was observed as well (see Table 4). For three participants, the perceived mortality risk between the best- and worst-case survival patients only differed by 20–30 percentage points. For five others, this difference was 60–80 percentage points.

### 3.5. Participants’ Feedback

In total, nine participants provided feedback on what information they missed in the vignette descriptions. Two participants expressed that they did not need any additional information. The remaining participants expressed a wish for more clinical details, that is, patients’ pulmonary status, survival prognoses for metastatic cancer, mobility status, and the motivation behind high ASA scores. Additionally, participants expressed that aspects of real-life decision-making were lacking in the vignettes. For example, second opinions from geriatricians, anaesthesiologists and cardiologists could have helped in shaping a better treatment proposal. Furthermore, the nuances of being able to look patients in the eye and ask them and their relatives about their personal treatment preferences were deemed important in real-life decision-making as well.

## 4. Discussion

This paper reports on the first quantitative decision analysis of surgical residents’ and orthopaedic trauma surgeons’ P-NOM recommendations for hypothetical cases of frail geriatric hip fracture patients. The results showed that metastatic carcinoma, severe heart failure, end-stage renal failure and dementia had the strongest influence on their preferences to recommend P-NOM. While cancer, renal insufficiency and dementia were already identified as common comorbidities in non-operatively treated hip fracture patients [13], we are the first to quantify their impact on physicians’ treatment recommendations. Furthermore, we found that preferences for P-NOM generally increased with perceived mortality risks. These findings underline and confirm that comorbidities leading to increased mortality risk are some of the strongest indicators to favour P-NOM over surgery [8,66].

However, some of these findings were unexpected, given the a priori hypotheses. First, based on the power analysis with an assumed OR of 1.4, no significant effect was expected to be found for the influence of dementia. In fact, with an observed OR of 3.35, dementia appeared to have a substantially higher influence on preferences for P-NOM than hypothesised. Second, the estimated effect size of metastatic carcinoma appeared to be highly influenced by the specification of the informative prior. The a priori assumed OR of 2.5 was substantially smaller than the observed ORs of 4.42 (2.14–8.95) and 6.41 (2.43–16.78) for informative and noninformative priors, respectively. This gives rise to the question of whether the influence of these attributes was undervalued in the a priori hypotheses or whether participants overvalued these attributes.

In retrospect, we would like to plead for the former. The a priori assumed effect sizes of the attributes were solely estimated based on their prognostic value for 30-day mortality. Initially, the assumption was made that 30-mortality risk could function as a viable proxy to model the ORs in the vignette study, since the risk of early death is a leading argument to elect P-NOM according to the national guidelines [8]. However, 30-day mortality risk alone may not be sufficient to fully encompass the benefit of P-NOM, as it overlooks QoL considerations [9]. A previous study found that over 90% of the 271 surveyed healthcare providers expressed that a poor postoperative QoL prospect was a common reason for them to treat frail geriatric hip fracture patients non-operatively [66]. Hence, we may have undervalued the influence of dementia and metastatic carcinomas in the priors as QoL was not accounted for. To substantiate these claims, important QoL considerations for both conditions will be delineated.

First, it is increasingly acknowledged that dementia is a terminal condition [67,68,69] that necessitates palliative care assessments [70]. This necessity is particularly pronounced in the advanced stages of dementia, with inclinations towards self-neglect in the form of malnutrition due to dysphagia [67]. In end-of-life care for demented older adults, Dutch clinicians agree that forgoing artificial nutrition and hydration (ANH) could be good medical practice [71], as ANH prolongs patients’ lives at the expense of serious discomfort [72]. Hence, safeguarding the QoL of demented patients may in fact entail safeguarding a humane death. However, these circumstances may not be applicable to all demented hip fracture patients, but primarily to those with advanced dementia [18]. Nevertheless, since preoperative dementia is a well-known significant risk factor for postoperative delirium, surgery may accelerate patients’ cognitive decline [42,43,44]. With these outcomes in mind, the perceived benefit of P-NOM may come from poor postoperative QoL prognoses, on top of increased mortality risk.

Second, recovery-oriented surgery is unlikely to improve the well-being of geriatric hip fracture patients who are debilitated by advanced malignancy [6]. While pain reduction could be a viable reason to elect surgery [73], the treatment’s benefit depends on the patient’s age and health status. Preference studies have shown that cancer patients aged 65 years and older are less willing to trade prolonged survival for decreased QoL than their younger counterparts [74]. Especially for those who are frail and suffer from metastasis, the best supportive care could be preferred due to its acceptable outcomes with respect to QoL [75,76,77]. Therefore, considering the implications of frailty and patients’ end-of-life preferences, QoL aspects may have contributed to the perceived benefit of P-NOM for hip fracture patients with metastatic cancer.

While several patient attributes were found to be critical for preoperative decision-making, it should be noted that treatment preferences were rather heterogeneous. On the one hand, heterogeneity in stated preferences could be attributable to the simplified nature of the vignettes, leading to a lack of nuances, which could have helped participants assess the patient cases more confidently and reliably. On the other hand, even for vignettes where participants consistently expressed (high) certainty for the optimality of their treatment recommendations, stated preferences remained divided. These observations are most likely reflecting the lack of guidelines for considering P-NOM.

Besides that, substantial heterogeneity in 30-day mortality risk perceptions was observed as well. This exemplifies the need for objective 30-day mortality prediction models to streamline risk perceptions. Through the SEJ, an attempt was made to forge a rational consensus between participants’ dispersed risk estimates. Through linear opinion pooling with performance-based weights, the expert judgements yielded a 30-day mortality prediction range between 11.9 and 50.8% across all vignettes. However, the maximum risk estimate appeared to be rather low, considering that it was the prognosis for a male institutionalised ASA IV patient between the ages of 80 and 89 years with severe functional handicaps, severe heart failure, metastatic cancer and end-stage renal failure.

To place the expert-driven estimates into perspective, a comparison was made with data-driven prediction models. An overview of the maximum predicted risks and the respective predictor variables of the Nottingham Hip Fracture Score (NHFS) [22], Almelo Hip Fracture Score (AHFS) [20], AHFS^90^ [78] and Brabant Hip Fracture Score (BHFS) [21] is shown in Table 5. This overview shows that most predictors also appear in the vignettes. The vignettes, however, include three strong predictors for 30-day mortality that are not included in the prediction models: metastatic cancer, severe heart failure and end-stage renal failure. Based on our systematic review [35], we observed that these predictors have larger effect sizes than most of the other predictors considered in the NHFS, AHFS, AHFS^90^ and BHFS. Yet, the performance-based weights estimate only attained a marginally higher maximum risk than the NHFS and BHFS. In comparison to the AHFS and the AHFS^90^, physicians’ collective judgements were lower, regardless of using equal or performance-based weights. As the maximum AHFS and AHFS^90^ were computed in a relatively healthy population compared to the vignettes, physicians’ collective estimates are likely downward biassed for the most vulnerable patients. Thus, patients at high risk of early mortality are potentially underidentified in practice.

However, several limitations may have downward biassed the equal and performance-based weight estimates for the most vulnerable patients. Since only nine participants completed the SEJ, underestimations may have been observed due to chance. Replication of the study in a larger cohort is necessary to confirm the findings. Nevertheless, two well-calibrated surgical residents were observed in this sample, accounting for a cumulative weight of 93% in the pooled estimates. Based on the premise of the SEJ, it is counterintuitive that they underestimated the 30-day mortality risk for the most vulnerable patients. It is postulated that the calibration questions did not capture the relevant range of expertise for the diverse vignettes, as the questions’ true realisations were limited to 30-day mortality rates between 3.9 and 33.2%. As the SEJ instrument calibrated participants to relatively healthy patients, a high calibration score did not reflect accurate predictions for high-risk patients. Future researchers should examine how the limited data on high-risk patients can be used more effectively to develop representative calibration questions.

Another limitation of this study was that the multi-faceted decision context was solely represented by surgical residents and orthopaedic trauma surgeons. Recommending adequate palliative care remains a challenging task, as trade-offs are to be made between relieving pain through surgery with risks of iatrogenesis [10,11] and resorting to analgesics in P-NOM with higher risks of pain undertreatment [9]. In our study, the participants expressed that the perspectives of other clinicians, such as geriatricians and anaesthesiologists, could have helped in improving the adequacy of the treatment proposals. Affirmatively, studies have shown that consultations with geriatricians were highly influential in the preoperative decision-making process [18]. Hence, the expertise from a broader group of clinical stakeholders may be necessary to adequately develop guidelines for considering P-NOM.

Finally, the results of the vignette study should be interpreted with caution due to the small sample size. According to our a priori power analysis, the study was only sufficiently powered for a few attributes: metastatic carcinoma, severe heart failure and end-stage renal failure. Increasing the sample size will likely result in the detection of meaningful effects for physical status, preoperative residence and functional status, since the ORs of these attributes have 95% CrIs, which are close to non-overlapping with the null effect. As it may be challenging to enrol sufficient participants, our posterior distributions could be used as highly informative priors in future work. By systematically updating the evidence found in this study through a Bayesian framework, the foundations laid here may strongly alleviate large sample size requirements in future studies.

## 5. Conclusions

This study demonstrated that the presence of metastatic carcinomas (OR: 4.42, CrI: 2.14–8.95), severe heart failure (OR: 4.05, CrI: 1.89–8.29), end-stage renal failure (OR: 3.54, CrI: 1.76–7.35) and dementia (OR: 3.35, CrI: 1.70–7.06) had the strongest influence on the decisions of surgical residents and orthopaedic trauma surgeons to favour P-NOM in frail geriatric hip fracture patients. Although they were more inclined to abstain from surgery amongst patients for whom higher 30-day mortality risks were prognosticated on average, heterogeneity in treatment preferences and risk perceptions was substantial. Hence, objective 30-day mortality prediction models should be used in clinical practice to streamline risk perceptions. However, objective mortality risk estimates alone are postulated to be insufficient to identify eligible candidates for P-NOM. Although meta-analyses revealed that some of the examined attributes were of small-to-moderate prognostic value for 30-day mortality, surgical residents and orthopaedic trauma surgeons could still strongly associate them with favouring P-NOM. The increased impact of these attributes is presumably derived from poor postoperative QoL prognoses, in addition to increased 30-day mortality risk. Hence, based on the stated preferences, more emphasis may need to be put on QoL considerations in clinical guidelines, to adequately provide decision support for considering P-NOM.

## Figures and Tables

**Table 1:** Overview of attributes and their levels as presented to the participants in the vignettes. For each attribute level, the rationale behind the chosen definition is provided.

Attribute	Levels	Rationale
Age	The patient is in the age group 80–89 years The patient is 90 years or older	80 years was chosen as a lower bound, based on the average age of hip fracture patients. The cut-off between the two levels was based on the observation that complication risks and mortality rates differed significantly between octogenarians and nonagenarians [37].
Gender	The patient is female The patient is male	-
Fracture type	The patient has an undisplaced femoral neck fracture The patient has a displaced femoral neck fracture The patient has an extracapsular fracture	The invasiveness of the required surgical intervention differs between displaced and undisplaced femoral neck fractures. Most extracapsular fractures are treated with intramedullary nails in The Netherlands. Hence, extracapsular fractures were not further distinguished.
Physical status	The patient has severe systemic diseases without a constant threat to life (ASA III) The patient has severe systemic diseases with a constant threat to life (ASA IV)	It was anticipated that ASA I, II and V would not require decision support: all ASA I and II patients would be treated operatively [8], and all ASA V patients would be treated non-operatively.
Severe heart failure	The patient has no severe heart failure (LVEF ≥ 30%) The patient has severe heart failure (LVEF < 30%)	A moderate-to-severe reduction in left ventricular ejection fraction (LVEF) is congruent with both ASA III and IV [38,39]. The corresponding cut-off of <30% was based on [40].
Metastatic carcinoma	The patient has no metastatic carcinomas The patient has metastatic carcinomas	The presence of non-metastatic cancer only increases the 30-day mortality risk weakly [35]. Hence, patients without metastases were not further distinguished into cancer-free and non-metastatic cancer patients.
End-stage renal failure	The patient has no end-stage renal failure requiring dialysis The patient has end-stage renal failure requiring dialysis	The dialysis requirement complies with both the ASA III and ASA IV classifications [38,39]. Due to the high prevalence of renal failure amongst adults aged ≥80 years [41], no distinction was made between mild renal failure and the absence of renal failure.
Preoperative residence	The patient lived at home prior to admission The patient lived in a care institution prior to admission	-
Functional status *	The patient has no severe functional handicaps (Katz 3–6) The patient has severe functional handicaps (Katz 0–2)	Low pre-fracture functioning was a common reason for choosing non-operative treatment [9,18]. Hence, the extreme end of the Katz scale was chosen.
Dementia	The patient has no dementia The patient has dementia	Dementia is a well-known predictor of postoperative delirium [42,43,44]. A single level for dementia was thus thought to be sufficient to influence clinicians’ decisions.

* Participants were provided with a link to a document where the Katz scores were explained in more detail, where 0 was defined as completely dependent and 6 as functionally independent.

**Table 2:** Overview of prior specifications expressed on a logarithmic scale. All betas denote log odds ratios (ORs).

Parameter	Distribution	Specification	Prior Type	Background Knowledge
β_0_	Normal	N(−2.75, 1)	Weakly informative	Here, 3% of the Dutch patients are treated non-operatively [56]. As the vignettes exclude ASA I-II, β_0_ was expected to be slightly higher. The prior yields a mean probability of 6.0% (95% CrI: 0.9–31.2%) in favour of P-NOM for the null model.
β_gender_	Normal	N(0.09, 1)	Weakly informative	Male gender is a high-quality evidence predictor for 30-day mortality [35]. However, it was deemed unlikely that this would be reflected in participants’ treatment preferences. Hence, the informativeness of the prior was decreased, yielding a mean OR of 1.1 (95% CrI: 0.15–7.80) in favour of P-NOM.
β_extracapsular_	Normal	N(0.09, 1)	Weakly informative	Compared to undisplaced femoral neck fractures, extracapsular fractures have a higher postoperative anaemia incidence [57]. Due to the lack of strong evidence for increased mortality risk [58], a small mean OR of 1.1 (95% CrI: 0.15–7.80) in favour of P-NOM was assumed.
β_DFN_	Normal	N(0.18, 1)	Weakly informative	Displaced femoral neck fractures require more invasive surgical intervention than their undisplaced counterparts. As quantitative evidence was lacking, a small mean OR of 1.2 (95% CrI: 0.17–8.51) in favour of P-NOM was assumed.
β_ASA_	Normal	N(0.69, 1)	Informative	ASA scores increase 30-day mortality risk with an OR of 2.62 (95% CI: 2.21–3.12) per point increase [35]. During the vignette study pilot test, a surgical resident expressed indifference towards ASA scores due to the subjectivity of the scoring system. A relatively wide prior was chosen to reflect uncertainty in the influence of ASA scores, with a mean OR of 2.0 (95% CrI: 0.5–7.99) in favour of P-NOM.
β_heart_	Normal	N(0.69, 0.5)	Informative	Heart failure increases the risk of 30-day mortality with an OR of 2.18 (95% CI: 1.25–3.82) [35]. The prior yields a mean OR of 2.0 (95% CrI: 0.50–7.98) in favour of P-NOM.
β_metastasis_	Normal	N(0.92, 0.3)	Informative	Metastasis increases the 30-day mortality risk with an OR of 2.83 (95% CI: 2.58–3.10) [35]. The informativeness of the prior was increased due to the high quality of the evidence and the narrow CI width. The prior yields a mean OR of 2.5 (95% CrI: 0.85–7.32) in favour of P-NOM.
β_ESRF_	Normal	N(0.79, 0.5)	Informative	Chronic renal failure increases the risk of 30-day mortality with an OR of 1.61 (95% CI: 1.11–2.34) [35]. The risk is even higher for ESRF (95% CI: 3.57–12.58) [59]. The prior yields a mean OR of 2.2 (95% CrI: 0.55–8.81) in favour of P-NOM.
β_institution_	Normal	N(0.47, 0.5)	Informative	Institutional residence increases the risk of 30-day mortality with an OR of 1.81 (95% CI: 1.31–2.49) [35]. The prior yields a mean OR of 1.6 (95% CrI: 0.40–6.42) in favour of P-NOM.
β_functional_	Normal	N(0.47, 0.7)	Informative	The effect size of severe functional handicaps was assumed to be similar to that of institutional residence. However, due to the lack of quantitative evidence, a slightly wider prior was specified with a mean OR of 1.6 (95% CrI: 0.31–8.26).
β_dementia_	Normal	N(0.34, 0.5)	Informative	Dementia increases the risk of 30-day mortality with an OR of 1.57 (95% CI: 1.30–1.90) [35]. The prior yields a mean OR of 1.4 (95% CrI: 0.35–5.60) in favour of P-NOM.
u_i_	Normal	N(0, σ^2^_u_)	Uninformative	N/A
σ^2^_u_	Inverse Wishart	IW(1, 1)	Uninformative	N/A
ε_i,j_	Multivariate normal	MVN(0, I) ^1^	Uninformative	N/A

DFN displaced femoral neck fracture, ASA American Society of Anesthesiologists physical status classification, ESRF end-stage renal failure, u_i_ random intercept term for a single participant, σ^2^_u_ variance of u_i_, ε_i,j_ random error term for a single participant and vignette, CrI credible interval. ^1^ Multivariate normal distribution with the mean vector equal to the zero vector and the covariance matrix equal to the identity matrix.

**Table 3:** Influence of patient characteristics on preferences for palliative non-operative management. Differences in odds ratios due to prior assumptions are quantified as relative deviations.

Attribute	Level	Informative Priors		Noninformative Priors		Deviation
		Odds Ratio	95% CrI	Odds Ratio	95% CrI	
Metastatic carcinoma	Present	4.42	**2.14–8.95**	6.41	**2.43–16.78**	45.0%
	Absent *					
Severe heart failure	Present	4.05	**1.89–8.29**	4.72	**2.13–11.48**	16.5%
	Absent *					
End-stage renal failure	Present	3.54	**1.76–7.35**	3.70	**1.56–8.86**	4.5%
	Absent *					
Dementia	Present	3.35	**1.70–7.06**	3.96	**1.75–10.03**	18.2%
	Absent *					
Physical status	ASA IV	1.92	0.92–4.37	1.49	0.63–3.84	22.4%
	ASA III *					
Preoperative residence	Institution	1.85	0.97–3.59	1.90	0.81–4.36	2.7%
	Home *					
Functional status	Severe handicaps	1.71	0.83–3.49	1.65	0.72–3.81	3.5%
	No severe handicaps *					
Gender	Male	1.55	0.74–3.42	1.53	0.67–3.58	1.3%
	Female *					
Age	≥90 years	1.20	0.54–2.59	1.12	0.49–2.56	6.7%
	80–89 years *					
Fracture type	Displaced femoral neck	1.00	0.41–2.41	0.85	0.32–2.34	15.0%
	Extracapsular	0.81	0.34–1.92	0.67	0.26–1.83	17.3%
	Undisplaced femoral neck *					

* Reference level. The 95% CrIs displayed in bold are strictly non-overlapping with the null effect.

**Table 4:** Summary of responses to the vignette study and structured expert judgement on participant level. Responses have been summarised across all 16 vignettes, with the risk range referring to the mortality risk for the best- and worst-case survival patients as estimated by the orthopaedic trauma surgeon/surgical resident.

Profession	Experience	P-NOM Recommendations	Risk Range
Orthopaedic trauma surgeon	>10 years	12/16 vignettes	35–95%
Orthopaedic trauma surgeon	>10 years	10/16 vignettes	25–65%
Orthopaedic trauma surgeon	>10 years	4/16 vignettes	35–75%
Orthopaedic trauma surgeon	5–10 years	12/16 vignettes	15–65%
Orthopaedic trauma surgeon	5–10 years	11/16 vignettes	5–35%
Orthopaedic trauma surgeon	5–10 years	3/16 vignettes	15–55%
Surgical resident	5–10 years	7/16 vignettes	15–85%
Surgical resident	5–10 years	3/16 vignettes	5–55%
Surgical resident	5–10 years	2/16 vignettes	5–65%
Surgical resident	<5 years	8/16 vignettes	15–55%
Surgical resident	<5 years	4/16 vignettes	15–95%
Surgical resident	<5 years	2/16 vignettes	65–95%
Surgical resident	<5 years	2/16 vignettes	15–75%
Surgical resident	<5 years	0/16 vignettes	15–35%

**Table 5:** Comparison of maximum 30-day mortality risks as estimated by prediction models and physicians’ judgements. Attributes included in prediction models/vignette are marked with an X.

Attribute	30-Day Mortality Prediction Models				Physicians	
	NHFS (45.0%)	AHFS (68.4%)	AHFS^90^ (64.5%)	BHFS (46.6%)	EW (62.9%)	PW (50.8%)
Age	X	X	X	X	X	X
Gender	X	X	X	X	X	X
Preoperative residence	X	X	X	X	X	X
History of malignancy	X	X		X	X ^1^	X ^1^
Cognitive impairment	X	X	X		X	X
Admission haemoglobin	X	X	X	X		
ASA classification		X	X		X	X
Number of comorbidities	X	X				
Mobility		X				
COPD				X		
Diabetes				X		
Functional status					X	X
Severe heart failure					X	X
End-stage renal failure					X	X
Fracture type					X	X

NHFS Nottingham Hip Fracture Score, AHFS Almelo Hip Fracture Score, AHFS^90^ Almelo Hip Fracture Score in patients aged ≥90 years, BHFS Brabant Hip Fracture Score, EW equally weighted pooled estimate, PW performance-weighted pooled estimate, ASA American Society of Anesthesiologists physical status classification, COPD chronic obstructive pulmonary disease. ^1^ Malignancy was exclusively defined as metastatic cancer.

**Figure A1:**
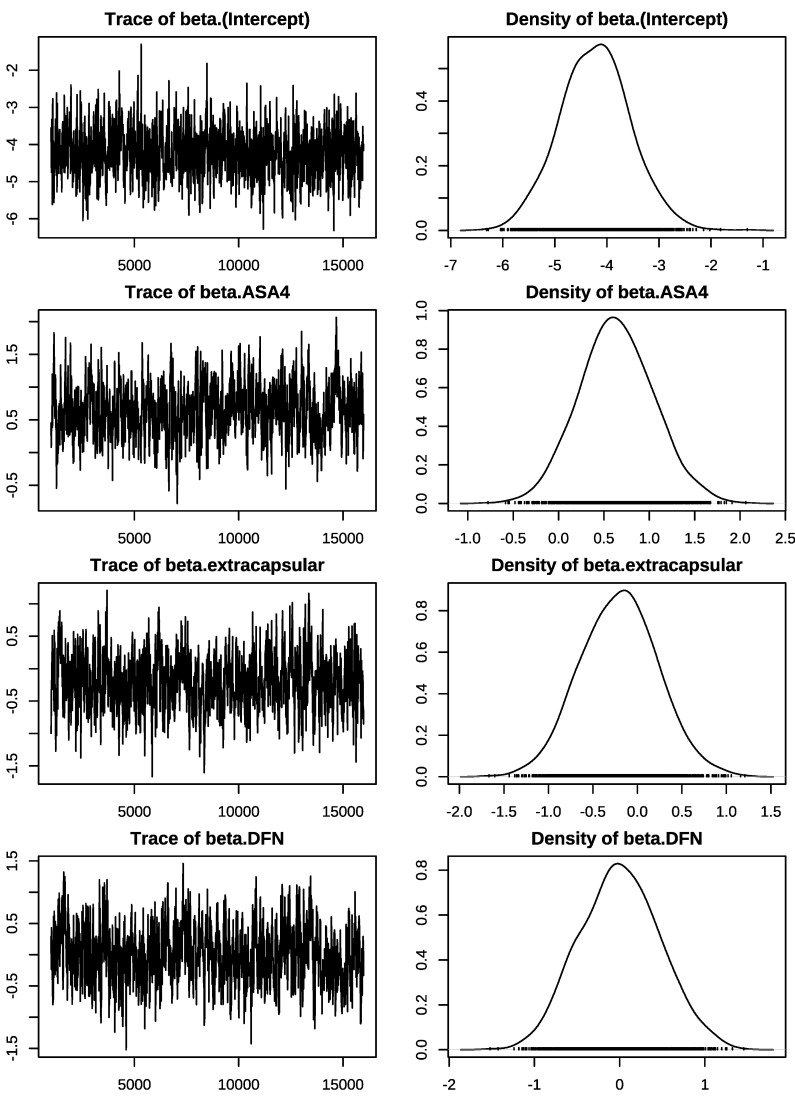
Trace plots and densities of the posterior distributions of the coefficients (1/3).

**Figure A2:**
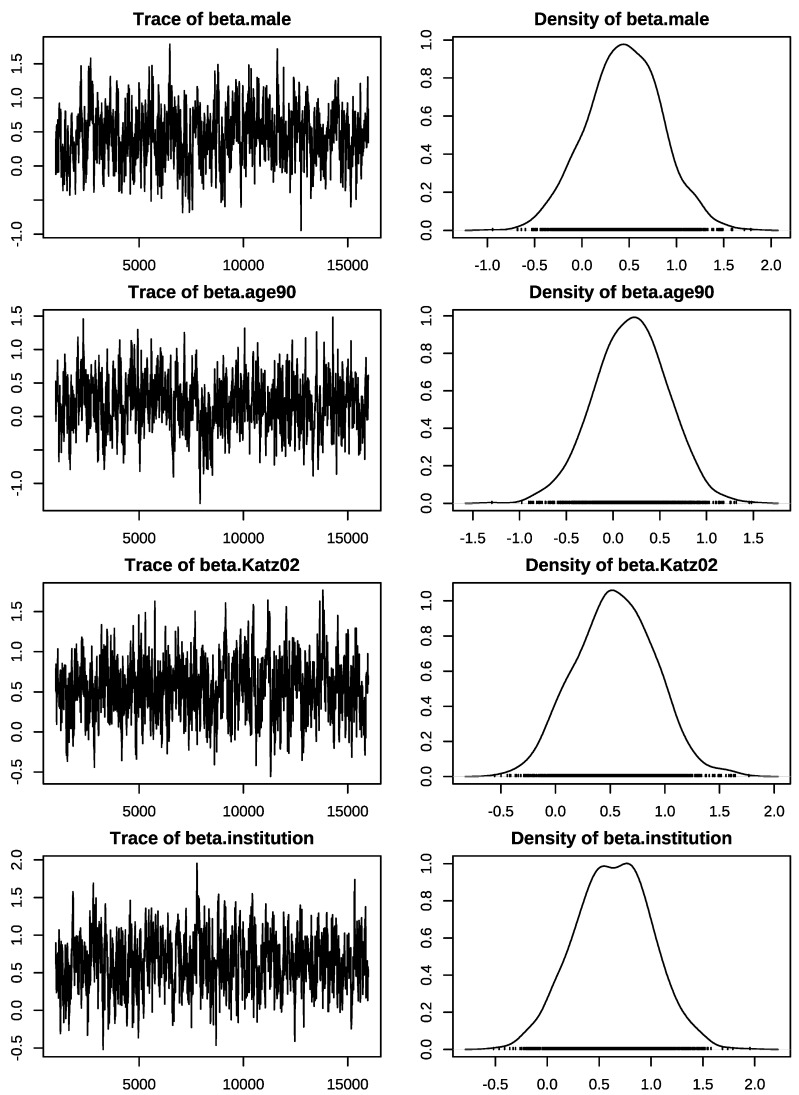
Trace plots and densities of the posterior distributions of the coefficients (2/3).

**Figure A3:**
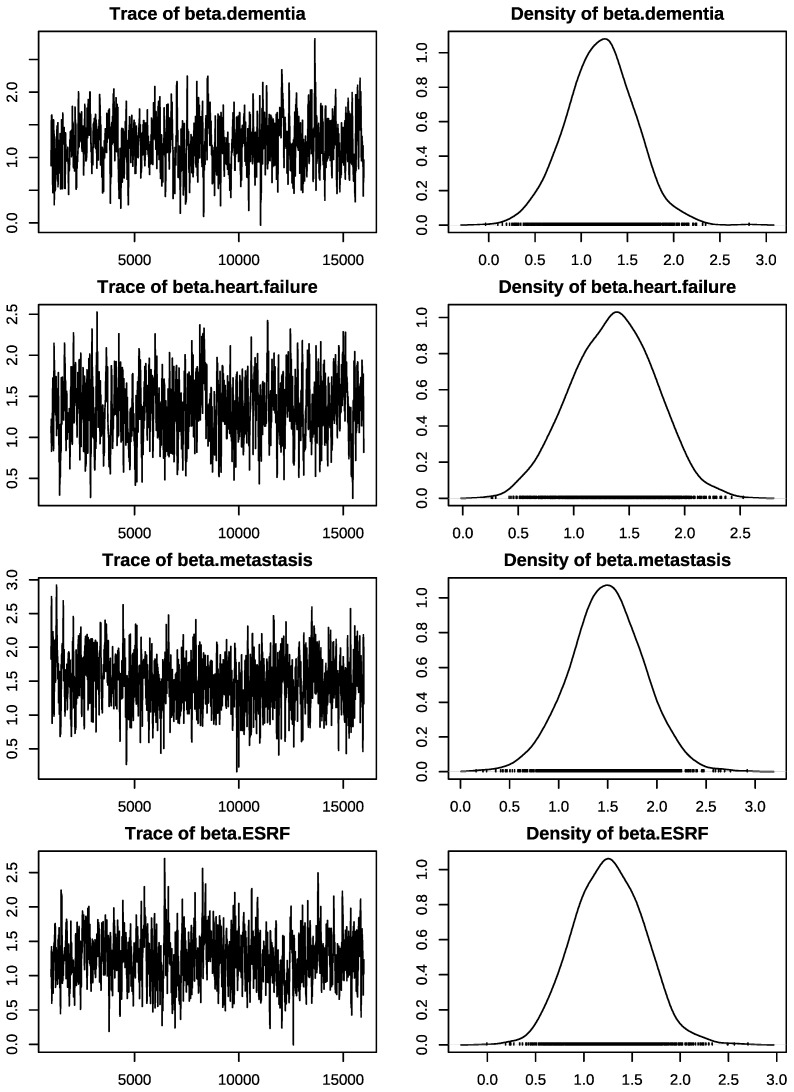
Trace plots and densities of the posterior distributions of the coefficients (3/3).

**Figure A4:**
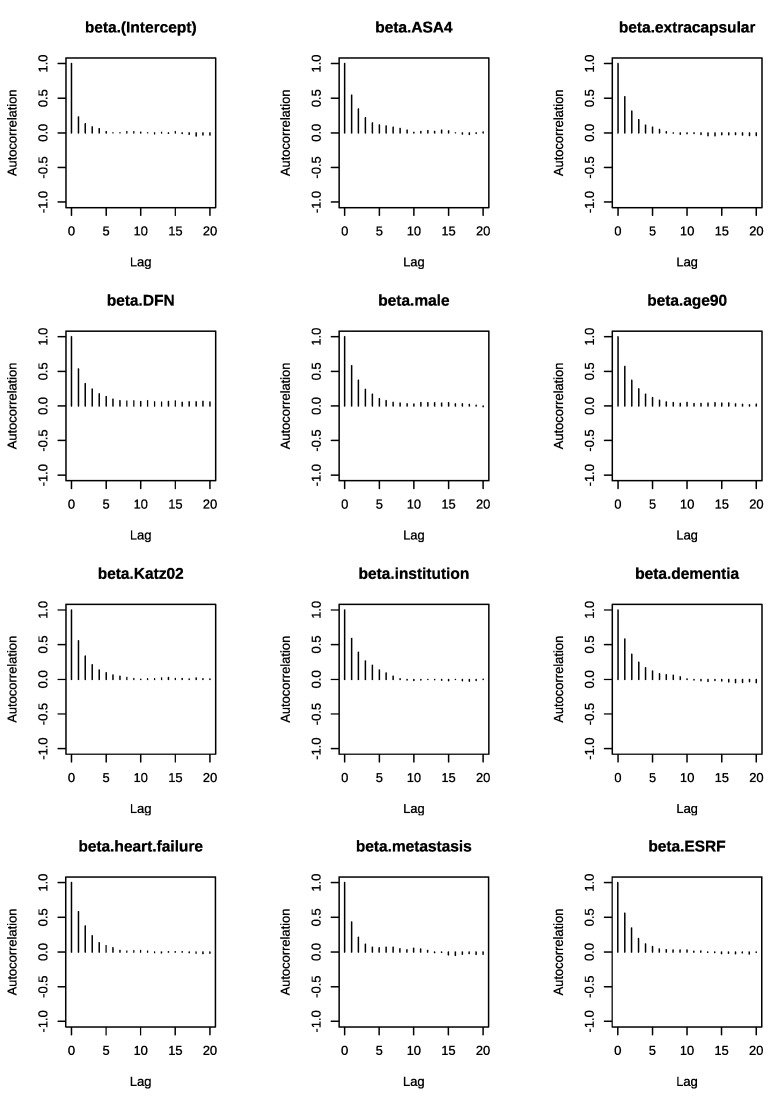
Autocorrelation plots of the Markov chains for each regression coefficient.

**Figure A5:**
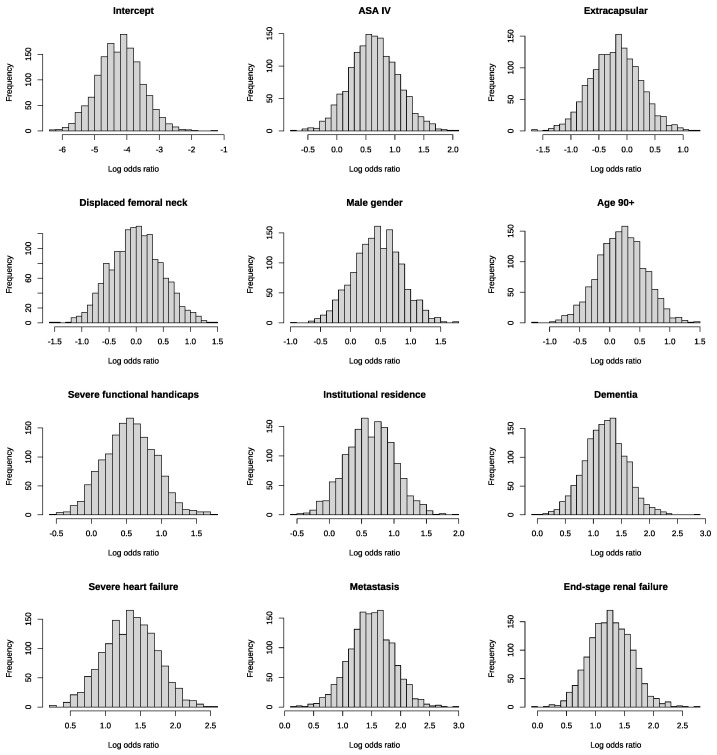
Histograms of the posterior samples drawn through Markov Chain Monte Carlo sampling for each regression coefficient.

## Data Availability

The data are not available as no consent was obtained from the participants to share the raw data.

## Appendix A

**Table A1:** Complete descriptions of the 16 vignettes included in the experimental design.

ID	ASA	Fracture	Gender	Age	Severe Functional Handicaps	Residence	Dementia	Severe Heart Failure	Metastasis	ESRF
1	III	DFN	Female	80–89	No	Home	No	No	No	No
2	IV	UFN	Female	90+	No	Home	No	Yes	No	Yes
3	IV	DFN	Female	90+	No	Institution	Yes	No	Yes	Yes
4	IV	EXT	Male	80–89	Yes	Institution	No	Yes	Yes	Yes
5	IV	EXT	Female	90+	Yes	Home	No	No	Yes	No
6	III	DFN	Male	80–89	Yes	Home	Yes	No	No	Yes
7	IV	UFN	Female	80–89	Yes	Institution	Yes	No	No	Yes
8	IV	UFN	Male	80–89	No	Home	Yes	Yes	Yes	No
9	IV	DFN	Male	90+	Yes	Home	Yes	Yes	Yes	No
10	III	EXT	Female	90+	Yes	Home	Yes	Yes	Yes	Yes
11	IV	DFN	Female	80–89	Yes	Institution	No	Yes	Yes	No
12	III	DFN	Male	90+	No	Institution	No	Yes	Yes	Yes
13	III	UFN	Male	90+	Yes	Institution	No	No	Yes	No
14	IV	EXT	Male	80–89	No	Home	No	No	Yes	Yes
15	IV	EXT	Male	90+	No	Institution	Yes	No	Yes	No
16	III	EXT	Female	80–89	No	Institution	Yes	Yes	Yes	No

ASA American Society of Anesthesiologists physical status classification, ESRF end-stage renal failure, DFN displaced femoral neck, UFN undisplaced femoral neck, EXT extracapsular.

**Table A2:** Overview of calibration questions and the corresponding 30-day mortality rates according to the Dutch Hip Fracture Audit Taskforce Indicators (DHFA-TF) group.

ID	Calibration Question	Mortality
1	How many percent of the **female** hip fracture patients aged **80 years or older** died within 30 days following hip fracture surgery between 2017 and 2019, according to the DHFA-TFI group?	8.0%
2	How many percent of the **male** hip fracture patients aged **90 years or older** died within 30 days following hip fracture surgery between 2017 and 2019, according to the DHFA-TFI group?	18.3%
3	How many percent of the hip fracture patients aged **85 years or older** with an **ASA IV classification** died within 30 days following hip fracture surgery between 2017 and 2019, according to the DHFA-TFI group?	25.9%
4	How many percent of the hip fracture patients aged **80 years or older** with an **ASA II-III classification** died within 30 days following hip fracture surgery between 2017 and 2019, according to the DHFA-TFI group?	8.2%
5	How many percent of the hip fracture patients aged **80 years or older** with a **high risk of malnutrition** (SNAQ score ≥ 3) and **pre-fracture institutional residence** died within 30 days following hip fracture surgery between 2017 and 2019, according to the DHFA-TFI group?	22.8%
6	How many percent of the hip fracture patients aged **80 years or older** with a **high risk of malnutrition** (SNAQ score ≥ 3) and **preoperative anaemia** died within 30 days following hip fracture surgery between 2017 and 2019, according to the DHFA-TFI group?	13.9%
7	How many percent of the hip fracture patients aged **80 years or older** with a **displaced femoral neck fracture** died within 30 days following hip fracture surgery between 2017 and 2019, according to the DHFA-TFI group?	6.5%
8	How many percent of the hip fracture patients aged **80 years or older** who were **fully independent in activities of daily living** (Katz score of 6) and at **low risk of malnutrition** (SNAQ score ≥ 1) died within 30 days following hip fracture surgery between 2017 and 2019, according to the DHFA-TFI group?	4.2%
9	How many percent of the hip fracture patients aged **90 years or older**, who were **mobile without walking aids** and **did not have dementia**, died within 30 days following hip fracture surgery between 2017 and 2019, according to the DHFA-TFI group?	10.2%
10	How many percent of the hip fracture patients aged **80 years or older** with an **ASA IV classification** and **prefracture institutional residence** died within 30 days following hip fracture surgery between 2017 and 2019, according to the DHFA-TFI group?	33.2%
11	How many percent of the hip fracture patients aged **90 years or older** with an **extracapsular fracture** and **preoperative anaemia** died within 30 days following hip fracture surgery between 2017 and 2019, according to the DHFA-TFI group?	15.7%
12	How many percent of the hip fracture patients aged **90 years or older** with an **ASA I-II classification** died within 30 days following hip fracture surgery between 2017 and 2019, according to the DHFA-TFI group?	3.9%
13	How many percent of the hip fracture patients aged **90 years or older** with an **ASA III-IV** classification, dementia and **pre-fracture institutional residence** died within 30 days following hip fracture surgery between 2017 and 2019, according to the DHFA-TFI group?	23.2%
14	How many percent of the hip fracture patients aged **90 years or older** with **severe functional handicaps** (Katz score 0–2) died within 30 days following hip fracture surgery between 2017 and 2019, according to the DHFA-TFI group?	16.7%

Katz and SNAQ scores were explained to the participants. Patient characteristics of interest were emphasised in bold to improve the readability of the calibration questions in the survey.

## Appendix B

Convergence of the Markov chains was confirmed visually by inspection of the trace plots (see Figure A1, Figure A2 and Figure A3) and autocorrelation plots (see Figure A4). The trendless trace plots and rapid decays in the autocorrelation plots were indicative of healthy convergence. Furthermore, Geweke’s convergence test yielded p-values above 0.05 for all regression coefficients, implying that stationarity could be assumed. Upon inspecting the frequency histograms of the posterior draws (see Figure A5), the distributions appeared to be smooth without substantial gaps between the bins. Hence, 15,000 samples were ample enough to adequately represent the posterior distributions.

## References

[1] Bhandari M., Swiontkowski M. (2017). Management of Acute Hip Fracture. N. Engl. J. Med..

[2] Parker M., Johansen A. (2006). Hip Fracture. BMJ.

[3] van de Ree C.L.P., De Jongh M.A.C., Peeters C.M.M., de Munter L., Roukema J.A., Gosens T. (2017). Hip Fractures in Elderly People: Surgery or No Surgery? A Systematic Review and Meta-Analysis. Geriatr. Orthop. Surg. Rehabil..

[4] Kim S.-J., Park H.-S., Lee D.-W. (2020). Outcome of Nonoperative Treatment for Hip Fractures in Elderly Patients: A Systematic Review of Recent Literature. J. Orthop. Surg..

[5] Berry S.D., Rothbaum R.R., Kiel D.P., Lee Y., Mitchell S.L. (2018). Association of Clinical Outcomes with Surgical Repair of Hip Fracture vs Nonsurgical Management in Nursing Home Residents with Advanced Dementia. JAMA Intern. Med..

[6] McNamara P., Sharma K. (1997). Surgery or Palliation for Hip Fractures in Patients with Advanced Malignancy?. Age Ageing.

[7] Cannada L.K., Mears S.C., Quatman C. (2021). Clinical Faceoff: When Should Patients 65 Years of Age and Older Have Surgery for Hip Fractures, and When Is It a Bad Idea?. Clin. Orthop. Relat. Res..

[8] Federatie Medisch Specialisten Richtlijn Proximale Femurfracturen. https://richtlijnendatabase.nl/richtlijn/behandeling_kwetsbare_ouderen_bij_chirurgie/proximale_femurfractuur_preoperatieve_traject/besluitvorming_bij_proximale_femurfractuur.html.

[9] Loggers S.A.I., Willems H.C., Van Balen R., Gosens T., Polinder S., Ponsen K.J., Van de Ree C.L.P., Steens J., Verhofstad M.H.J., Zuurmond R.G. (2022). Evaluation of Quality of Life after Nonoperative or Operative Management of Proximal Femoral Fractures in Frail Institutionalized Patients: The FRAIL-HIP Study. JAMA Surg..

[10] Clapp J.T., Schwarze M.L., Fleisher L.A. (2022). Surgical Overtreatment and Shared Decision-Making—The Limits of Choice. JAMA Surg..

[11] Cardona-Morrell M., Kim J., Turner R.M., Anstey M., Mitchell I.A., Hillman K. (2016). Non-Beneficial Treatments in Hospital at the End of Life: A Systematic Review on Extent of the Problem. Int. J. Qual. Health Care.

[12] Nijdam T.M.P., Laane D.W.P.M., Spierings J.F., Schuijt H.J., Smeeing D.P.J., van der Velde D. (2022). Proxy-Reported Experiences of Palliative, Non-Operative Management of Geriatric Patients after a Hip Fracture: A Qualitative Study. BMJ Open.

[13] Loggers S.A.I., Van Lieshout E.M.M., Joosse P., Verhofstad M.H.J., Willems H.C. (2020). Prognosis of Nonoperative Treatment in Elderly Patients with a Hip Fracture: A Systematic Review and Meta-Analysis. Injury.

[14] Wijnen H.H., Schmitz P.P., Es-Safraouy H., Roovers L.A., Taekema D.G., Van Susante J.L.C. (2021). Nonoperative Management of Hip Fractures in Very Frail Elderly Patients May Lead to a Predictable Short Survival as Part of Advance Care Planning. Acta Orthop..

[15] Ko F.C., Morrison R.S. (2014). Hip Fracture: A Trigger for Palliative Care in Vulnerable Older Adults. JAMA Intern. Med..

[16] Neuman M.D., Silber J.H., Magaziner J.S., Passarella M.A., Mehta S., Werner R.M. (2014). Survival and Functional Outcomes after Hip Fracture among Nursing Home Residents. JAMA Intern. Med..

[17] Joosten E.a.G., DeFuentes-Merillas L., de Weert G.H., Sensky T., van der Staak C.P.F., Jong C.A.J. (2008). de Systematic Review of the Effects of Shared Decision-Making on Patient Satisfaction, Treatment Adherence and Health Status. Psychother. Psychosom..

[18] van der Zwaard B.C., Stein C.E., Bootsma J.E.M., van Geffen H.J.A.A., Douw C.M., Keijsers C.J.P.W. (2020). Fewer Patients Undergo Surgery When Adding a Comprehensive Geriatric Assessment in Older Patients with a Hip Fracture. Arch. Orthop. Trauma. Surg..

[19] Prommik P., Tootsi K., Saluse T., Märtson A., Kolk H. (2021). Nonoperative Hip Fracture Management Practices and Patient Survival Compared to Surgical Care: An Analysis of Estonian Population-Wide Data. Arch. Osteoporos..

[20] Nijmeijer W.S., Folbert E.C., Vermeer M., Slaets J.P., Hegeman J.H. (2016). Prediction of Early Mortality Following Hip Fracture Surgery in Frail Elderly: The Almelo Hip Fracture Score (AHFS). Injury.

[21] van de Ree C.L., Gosens T., van der Veen A.H., Oosterbos C.J., Heymans M.W., de Jongh M.A. (2020). Development and Validation of the Brabant Hip Fracture Score for 30-Day and 1-Year Mortality. HIP Int..

[22] Moppett I.K., Parker M., Griffiths R., Bowers T., White S.M., Moran C.G. (2012). Nottingham Hip Fracture Score: Longitudinal and Multi-Centre Assessment. Br. J. Anaesth..

[23] Yenidogan B., Pathak S., Geerdink J., Hegeman J.H., van Keulen M. Multimodal Machine Learning for 30-Days Post-Operative Mortality Prediction of Elderly Hip Fracture Patients. Proceedings of the 2021 International Conference on Data Mining Workshops (ICDMW).

[24] Cary M.P., Zhuang F., Draelos R.L., Pan W., Amarasekara S., Douthit B.J., Kang Y., Colón-Emeric C.S. (2021). Machine Learning Algorithms to Predict Mortality and Allocate Palliative Care for Older Patients with Hip Fracture. J. Am. Med. Dir. Assoc..

[25] French S., Hanea A.M., Bedford T., Nane G.F., Hanea A.M. (2021). Introduction and Overview of Structured Expert Judgement. Expert Judgement in Risk and Decision Analysis.

[26] Hanea A.M., Nane G.F. (2019). Calibrating Experts’ Probabilistic Assessments for Improved Probabilistic Predictions. Saf. Sci..

[27] Quinn R.H., Mooar P.A., Murray J.N., Pezold R., Sevarino K.S. (2017). Treatment of Hip Fractures in the Elderly. J. Am. Acad. Orthop. Surg..

[28] Ryan M., Farrar S. (2000). Using Conjoint Analysis to Elicit Preferences for Health Care. BMJ.

[29] Ryan M., Scott D.A., Reeves C., Bate A., van Teijlingen E.R., Russell E.M., Napper M., Robb C.M. (2001). Eliciting Public Preferences for Healthcare: A Systematic Review of Techniques. Health Technol. Assess..

[30] Bachmann L.M., Mühleisen A., Bock A., ter Riet G., Held U., Kessels A.G. (2008). Vignette Studies of Medical Choice and Judgement to Study Caregivers’ Medical Decision Behaviour: Systematic Review. BMC Med. Res. Methodol..

[31] Atzmüller C., Steiner P.M. (2010). Experimental Vignette Studies in Survey Research. Methodology.

[32] Evans S.C., Roberts M.C., Keeley J.W., Blossom J.B., Amaro C.M., Garcia A.M., Stough C.O., Canter K.S., Robles R., Reed G.M. (2015). Vignette Methodologies for Studying Clinicians’ Decision-Making: Validity, Utility, and Application in ICD-11 Field Studies. Int. J. Clin. Health Psychol..

[33] Taylor B.J. (2006). Factorial Surveys: Using Vignettes to Study Professional Judgement. Br. J. Soc. Work.

[34] Cooke R.M. (1991). Experts in Uncertainty: Opinion and Subjective Probability in Science.

[35] Bui M., Nijmeijer W.S., Hegeman J.H., Witteveen A., Groothuis-Oudshoorn C.G.M. (2023). Systematic Review and Meta-Analysis of Preoperative Predictors for Early Mortality Following Hip Fracture Surgery. Osteoporos. Int..

[36] Daabiss M. (2011). American Society of Anaesthesiologists Physical Status Classification. Indian J. Anaesth..

[37] de Groot R., Nijmeijer W.S., Folbert E.C., Vollenbroek-Hutten M.M.R., Hegeman J.H. (2020). ‘Nonagenarians’ with a Hip Fracture: Is a Different Orthogeriatric Treatment Strategy Necessary?. Arch. Osteoporos..

[38] Hurwitz E.E., Simon M., Vinta S.R., Zehm C.F., Shabot S.M., Minhajuddin A., Abouleish A.E. (2017). Adding Examples to the ASA-Physical Status Classification Improves Correct Assignment to Patients. Anesthesiology.

[39] Mayhew D., Mendonca V., Murthy B.V.S. (2019). A Review of ASA Physical Status—Historical Perspectives and Modern Developments. Anaesthesia.

[40] Sweitzer N.K., Lopatin M., Yancy C.W., Mills R.M., Stevenson L.W. (2008). Comparison of Clinical Features and Outcomes of Patients Hospitalized with Heart Failure and Normal Ejection Fraction (> or =55%) versus Those with Mildly Reduced (40% to 55%) and Moderately to Severely Reduced (<40%) Fractions. Am. J. Cardiol..

[41] Stevens L.A., Viswanathan G., Weiner D.E. (2010). Chronic Kidney Disease and End-Stage Renal Disease in the Elderly Population: Current Prevalence, Future Projections, and Clinical Significance. Adv. Chronic Kidney Dis..

[42] Smith T.O., Cooper A., Peryer G., Griffiths R., Fox C., Cross J. (2017). Factors Predicting Incidence of Post-Operative Delirium in Older People Following Hip Fracture Surgery: A Systematic Review and Meta-Analysis. Int. J. Geriatr. Psychiatry.

[43] Bitsch M., Foss N., Kristensen B., Kehlet H. (2004). Pathogenesis of and Management Strategies for Postoperative Delirium after Hip fractureA Review. Acta Orthop. Scand..

[44] Mosk C.A., Mus M., Vroemen J.P., van der Ploeg T., Vos D.I., Elmans L.H., van der Laan L. (2017). Dementia and Delirium, the Outcomes in Elderly Hip Fracture Patients. Clin. Interv. Aging.

[45] Araujo B.L.d.C., Theobald D. (2017). Letter to the Editor: ASA Physical Status Classification in Surgical Oncology and the Importance of Improving Inter-Rater Reliability. J. Korean Med. Sci..

[46] Johnson F.R., Lancsar E., Marshall D., Kilambi V., Mühlbacher A., Regier D.A., Bresnahan B.W., Kanninen B., Bridges J.F.P. (2013). Constructing Experimental Designs for Discrete-Choice Experiments: Report of the ISPOR Conjoint Analysis Experimental Design Good Research Practices Task Force. Value Health.

[47] de Aguiar P.F., Bourguignon B., Khots M.S., Massart D.L., Phan-Than-Luu R. (1995). D-Optimal Designs. Chemom. Intell. Lab. Syst..

[48] Morgan-Wall T., Khoury G. (2021). Optimal Design Generation and Power Evaluation in R: The Skpr Package. J. Stat. Softw..

[49] Bridges J.F.P., Hauber A.B., Marshall D., Lloyd A., Prosser L.A., Regier D.A., Johnson F.R., Mauskopf J. (2011). Conjoint Analysis Applications in Health—A Checklist: A Report of the ISPOR Good Research Practices for Conjoint Analysis Task Force. Value Health.

[50] Ali A., Ali S., Khan S.A., Khan D.M., Abbas K., Khalil A., Manzoor S., Khalil U. (2019). Sample Size Issues in Multilevel Logistic Regression Models. PLoS ONE.

[51] McNeish D. (2016). On Using Bayesian Methods to Address Small Sample Problems. Struct. Equ. Model..

[52] Smid S.C., McNeish D., Miočević M., van de Schoot R. (2020). Bayesian Versus Frequentist Estimation for Structural Equation Models in Small Sample Contexts: A Systematic Review. Struct. Equ. Model..

[53] Regier D.A., Ryan M., Phimister E., Marra C.A. (2009). Bayesian and Classical Estimation of Mixed Logit: An Application to Genetic Testing. J. Health Econ..

[54] Miočević M., Levy R., Schoot R. (2020). van de Introduction to Bayesian Statistics. Small Sample Size Solutions.

[55] Depaoli S., van de Schoot R. (2017). Improving Transparency and Replication in Bayesian Statistics: The WAMBS-Checklist. Psychol. Methods.

[56] Aspinall W., Mader H., Coles S., Connor C., Connor L. (2006). Structured Elicitation of Expert Judgment for Probabilistic Hazard and Risk Assessment in Volcanic Eruptions. Statistics in Volcanology.

[57] Würdemann F.S., Wilschut J.A., Hegeman J.H. (2021). Eindverslag SKMS Project Doorontwikkeling DHFA.

[58] van Buuren S., Groothuis-Oudshoorn K. (2011). Mice: Multivariate Imputation by Chained Equations in R. J. Stat. Softw..

[59] Hanea A.M., Nane G.F., Hanea A.M., Nane G.F., Bedford T., French S. (2021). An In-Depth Perspective on the Classical Model. Expert Judgement in Risk and Decision Analysis.

[60] Gilks W.R., Armitage P., Colton T. (2005). Markov Chain Monte Carlo. Encyclopedia of Biostatistics.

[61] Martin A.D., Quinn K.M., Park J.H. (2011). MCMCpack: Markov Chain Monte Carlo in R. J. Stat. Softw..

[62] Geweke J., In F. (1995). Evaluating the Accuracy of Sampling-Based Approaches to the Calculation of Posterior Moments. https://www.researchgate.net/publication/2352607_Evaluating_the_Accuracy_of_Sampling-Based_Approaches_to_the_Calculation_of_Posterior_Moments.

[63] de Bekker-Grob E.W., Donkers B., Jonker M.F., Stolk E.A. (2015). Sample Size Requirements for Discrete-Choice Experiments in Healthcare: A Practical Guide. Patient.

[64] Harrison R.L. (2010). Introduction to Monte Carlo Simulation. AIP Conf. Proc..

[65] Marti D., Mazzuchi T.A., Cooke R.M., Hanea A.M., Nane G.F., Bedford T., French S. (2021). Are Performance Weights Beneficial? Investigating the Random Expert Hypothesis. Expert Judgement in Risk and Decision Analysis.

[66] Spronk I., Loggers S.A.I., Joosse P., Willems H.C., Van Balen R., Gosens T., Ponsen K.J., Steens J., Van de Ree L.C.P., Zuurmond R.G. (2022). Shared Decision-Making for the Treatment of Proximal Femoral Fractures in Frail Institutionalised Older Patients: Healthcare Providers’ Perceived Barriers and Facilitators. Age Ageing.

[67] Sachs G.A., Shega J.W., Cox-Hayley D. (2004). Barriers to Excellent End-of-Life Care for Patients with Dementia. J. Gen. Intern. Med..

[68] Mitchell S.L., Kiely D.K., Hamel M.B. (2004). Dying with Advanced Dementia in the Nursing Home. Arch. Intern. Med..

[69] Ahronheim J.C., Morrison R.S., Baskin S.A., Morris J., Meier D.E. (1996). Treatment of the Dying in the Acute Care Hospital. Advanced Dementia and Metastatic Cancer. Arch. Intern. Med..

[70] Boyd K., Murray S.A. (2010). Recognising and Managing Key Transitions in End of Life Care. BMJ.

[71] Rurup M.L., Onwuteaka-Philipsen B.D., Pasman H.R.W., Ribbe M.W., van der Wal G. (2006). Attitudes of Physicians, Nurses and Relatives towards End-of-Life Decisions Concerning Nursing Home Patients with Dementia. Patient Educ. Couns..

[72] Volicer L., Rheaume Y., Brown J., Fabiszewski K., Brady R. (1986). Hospice Approach to the Treatment of Patients with Advanced Dementia of the Alzheimer Type. JAMA.

[73] Sullivan N.M., Blake L.E., George M., Mears S.C. (2019). Palliative Care in the Hip Fracture Patient. Geriatr. Orthop. Surg. Rehabil..

[74] Yellen S.B., Cella D.F., Leslie W.T. (1994). Age and Clinical Decision Making in Oncology Patients. J. Natl. Cancer Inst..

[75] Adamowicz K., Baczkowska-Waliszewska Z. (2020). Quality of Life during Chemotherapy, Hormonotherapy or antiHER2 Therapy of Patients with Advanced, Metastatic Breast Cancer in Clinical Practice. Health Qual. Life Outcomes.

[76] Falci C., Morello E., Droz J.P. (2009). Treatment of Prostate Cancer in Unfit Senior Adult Patients. Cancer Treat. Rev..

[77] Hind D., Wyld L., Reed M.W. (2007). Surgery, with or without Tamoxifen, vs Tamoxifen Alone for Older Women with Operable Breast Cancer: Cochrane Review. Br. J. Cancer.

[78] Nijmeijer W.S., Voorthuis B.J., Groothuis-Oudshoorn C.G.M., Würdemann F.S., van der Velde D., Vollenbroek-Hutten M.M.R., Hegeman J.H., on behalf of the Dutch Hip Fracture Audit Taskforce Indicators Group (2023). The Prediction of Early Mortality Following Hip Fracture Surgery in Patients Aged 90 Years and Older: The Almelo Hip Fracture Score 90 (AHFS90). Osteoporos. Int..

